# Stromal accumulation of chondroitin sulphate in mammary tumours of dogs

**DOI:** 10.1038/sj.bjc.6690529

**Published:** 1999-07

**Authors:** U Hinrichs, G R Rutteman, H Nederbragt

**Affiliations:** Department of Pathology, Faculty of Veterinary Medicine, Utrecht University, PB 80.158, Utrecht, TD, 3508, The Netherlands; Department of Clinical Sciences of Companion Animals, Faculty of Veterinary Medicine, Utrecht University, PB 80.154, Utrecht, TD, 3508, The Netherlands

**Keywords:** sulphated glycosaminoglycans, chondroitin sulphate, fibronectin, extracellular matrix, cartilage, canine mammary tumour

## Abstract

To contribute to the investigation of the composition of the extracellular matrix in epithelial tumours, mammary gland tissues of dogs (including tumours, hyperplasias and normal tissue as well as metastatic lesions in lymph nodes and lung) were studied histochemically and immunohistochemically for distribution of sulphated glycosaminoglycans (s-GAGs). The formaline-fixed tissue was stained by alcian blue at pH 5.8, using the ‘critical electrolyte concentration’ to study the degree of sulphation of s-GAGs. s-GAGs were characterized by degradation with enzymes and nitrous acid and by immunohistochemistry with two anti-chondroitin sulphate monoclonal antibodies. The light microscopic investigation of s-GAG deposits revealed a limited number of patterns of their distribution. The main s-GAGs found in the mammary gland tumours of dogs and in metastatic lesions were chondroitin sulphate (CS) and heparin/heparan sulphate (HEP/HS). CS accumulated in diffuse structures between epithelial cells as well as around clusters of tumour cells. The latter pattern, possibly representing a mesenchymal reaction to the tumour, was present in 74% of the tumours, and in 67% of these, highly sulphated CS was present. A diffuse accumulation of CS was present almost exclusively in complex and mixed tumours; because of the expression of the 3B3 epitope for CS in immature cartilage the spindle cells of complex tumours are argued to be the precursors of the cartilage in mixed tumours. HEP/HS was stored mainly in mast cells that were found in increased numbers in hyperplasias and tumours. By pretreatment of microscopic slides with chondroitinase AC or ABC immunostaining of fibronectin could be made possible in areas in which CS was abundantly present, suggesting that CS may mask fibronectin epitopes. It is concluded that CS with different degrees of sulphation is the most important s-GAG in the extracellular matrix of mammary tumours of dogs. CS and other s-GAGs accumulate at different sites and may have a different pathogenetic significance. © 1999 Cancer Research Campaign


*British Journal of Cancer* (1999) **80**(9), 1359–1365
© 1999 Cancer Research Campaign
Article no. bjoc.1998.0529

### Stromal accumulation of chondroitin sulphate in mammary tumours of dogs

**U Hinrichs[1,]*, GR Rutteman[2] and H Nederbragt[1]**

[1]Department of Pathology, Faculty of Veterinary Medicine, Utrecht University, PB 80.158, 3508 TD Utrecht, The Netherlands; [2]Department of Clinical Sciences of Companion Animals, Faculty of Veterinary Medicine, Utrecht University, PB 80.154, 3508 TD Utrecht, The Netherlands

**Summary** To contribute to the investigation of the composition of the extracellular matrix in epithelial tumours, mammary gland tissues of dogs (including tumours, hyperplasias and normal tissue as well as metastatic lesions in lymph nodes and lung) were studied histochemically and immunohistochemically for distribution of sulphated glycosaminoglycans (s-GAGs). The formaline-fixed tissue was stained by alcian blue at pH 5.8, using the 'critical electrolyte concentration' to study the degree of sulphation of s-GAGs. s-GAGs were characterized by degradation with enzymes and nitrous acid and by immunohistochemistry with two anti-chondroitin sulphate monoclonal antibodies. The light microscopic investigation of s-GAG deposits revealed a limited number of patterns of their distribution. The main s-GAGs found in the mammary gland tumours of dogs and in metastatic lesions were chondroitin sulphate (CS) and heparin/heparan sulphate (HEP/HS). CS accumulated in diffuse structures between epithelial cells as well as around clusters of tumour cells. The latter pattern, possibly representing a mesenchymal reaction to the tumour, was present in 74% of the tumours, and in 67% of these, highly sulphated CS was present. A diffuse accumulation of CS was present almost exclusively in complex and mixed tumours; because of the expression of the 3B3 epitope for CS in immature cartilage the spindle cells of complex tumours are argued to be the precursors of the cartilage in mixed tumours. HEP/HS was stored mainly in mast cells that were found in increased numbers in hyperplasias and tumours. By pretreatment of microscopic slides with chondroitinase AC or ABC immunostaining of fibronectin could be made possible in areas in which CS was abundantly present, suggesting that CS may mask fibronectin epitopes. It is concluded that CS with different degrees of sulphation is the most important s-GAG in the extracellular matrix of mammary tumours of dogs. CS and other s-GAGs accumulate at different sites and may have a different pathogenetic significance.

**Keywords:** sulphated glycosaminoglycans; chondroitin sulphate; fibronectin; extracellular matrix; cartilage; canine mammary tumour

The importance of the extracellular matrix (ECM) for cancer growth and proliferation (Iozzo, 1988) as well as for development and differentiation of normal cells such as mammary epithelium (Roskelley and Bissell, 1995) has been stressed.

Proteoglycans (PGs) are extracellular proteins, either deposited in the ECM or present as cell surface proteins. They contain different types and numbers of glycosaminoglycan (GAG) side-chains and therefore show a wide array of variations in composition and function (reviewed in Jackson et al, 1991). Well-known large ECM chondroitin sulphate (CS)-containing PGs are aggrecan, a PG of cartilage, and versican; small ECM PGs are found in connective tissue, such as biglycan (two GAG chains) and decorin (one GAG chain). The latter has been demonstrated to bind extracellular transforming growth factor-β (TGF-β) (Iozzo, 1997) and regulate the cell cyclus via p21 (Santra et al, 1997). A well-described heparan sulphate PG (HSPG) of the ECM is perlecan, present in the basement membrane; the cell surface HSPG syndecan plays a role in the binding of basic fibroblast growth factor (bFGF) (David, 1993). The receptor for TGF-β III is a HS/CSPG (Andres et al, 1989).

*Received 23 June 1998*
*Revised 3 November 1998*
*Accepted 9 December 1998*

*Correspondence to:* H Nederbragt

The distribution and composition of PGs in the tumours and their stroma may be changed considerably compared to the normal tissue. In different types of tumours accumulation of CSPGs has been observed (Alini and Losa, 1991; Nara et al, 1991) and although the interstitial PG versican may be one of them (Nara et al, 1997), most of these tumour PGs have not been identified. Increased levels of PGs and sulphated glycosaminoglycans (s-GAGs) have been described for a variety of tumours (Iozzo, 1987), including human breast tumours (Alini and Losa, 1991). Some biological functions of s-GAGs have been investigated (Jackson et al, 1991), but still most of the significance of their diversity in form and function remains unclear. Attached to collagen or free in the ECM, some are able to bind growth factors and influence growth of normal and transformed cells in vitro. As part of the cell surface, they are probably acting as matrix receptors.

In the present work, mammary tumours of dogs were investigated, because of their remarkable diversity with regard to the presence of ECM components, including cartilage and bone. The mammary tumours of dogs are classified histologically in simple, complex and mixed tumours (Hampe and Misdorp, 1974) of benign or malignant nature. In the simple tumours, only one type of epithelial cells is proliferating, whereas in complex tumours, spindle-shaped cells of myoepithelial origin are present next to

*Present address: Aussenstelle für Epidemiologie der Tierärztlichen Hochschule Hannover, Bueschelerstr. 9, 49456 Bakum, Germany luminal epithelial cells. Mixed tumours are characterized by the presence of tumourous epithelial cells as well as of tumourous mesenchymal cells, and often contain cartilage and bone.

Complex carcinomas of dogs, already known to be rich in GAGs (Palmer and Monlux, 1979) are reported to be less malignant compared to their simple counterparts (Misdorp and Hart, 1976). The absence of data on the presence of GAGs in relation to benign or malignant behaviour in spontaneous tumours prompted us to investigate in detail the distribution of s-GAGs in mammary tumours. Normal and hyperplastic mammary glands, as well as benign and malignant tumours of different types, were studied light microscopically. Tissues were stained with alcian blue combined with the 'critical electrolyte concentration' (CEC) (Scott and Dorling, 1965) and enzyme or nitrous acid degradation of specific GAGs. In addition, immunohistochemistry was performed, to detect CS in the tissue.

We show here that abnormal amounts and types of CS are predominant findings in mammary benign and malignant tumours of dogs.

### MATERIALS AND METHODS

Eighty-five tissue samples of mammary glands resected surgically or collected at necropsy derived from 67 dogs were investigated. Metastatic lesions in eight lungs and nine lymph nodes were also included. Tissues were fixed immediately after surgery or death in 4% buffered formaldehyde and embedded in paraffin. Fixation time was restricted to 24 h to prevent leakage of GAGs out of the tissue (TA Niewold and PCJ Tooten, unpublished observations). Sections were cut to 5-µm in thickness and serial sections were stained with haematoxylin and eosin (H&E), alcian blue pH 5.8 combined with different magnesium chloride concentrations, degrading enzymes and nitrous acid. Additional immunoperoxidase staining was performed to detect the GAG side-chains of CSPG and the 3B3 epitope in native CS. Tumours were classified from the H&E-stained sections according to the WHO classification (Hampe and Misdorp, 1974).

For alcian blue staining, tissue slides were placed overnight in 0.05% alcian blue 8GX solutions in 0.2 M acetate buffer pH 5.8 containing 0.25, 0.55, 0.75, or 1.0 M $MgCl_2$. Degradation of s-GAGs with chondroitinase ABC (from *Proteus vulgaris*) and chondroitinase AC (from *Flavobacterium heparinum*) (both Sigma, St Louis, MO, USA) and nitrous acid was performed (Niewold et al, 1991). Briefly, after deparaffinization, tissue sections were entirely covered with chondroitinase AC and ABC, respectively, in a concentration of 2 units per ml in 0.25 M Tris buffer pH 8 containing 0.18 M sodium chloride, 0.05% bovine serum albumin (BSA) and 0.1 M 6-amino-n-caproic acid (Sigma) and 5 mM benzamidine hydrochloric acid (Sigma) to inhibit protease activity. Tissue slides were placed at 37°C for 4 h. As a control, serial slides were covered with the same buffer without enzymes. For degradation of heparin (HEP) and HS, slides were put in a solution containing 5% $NaNO_2$ and 33% acetic acid mixed 1:1 that was allowed to stand for 90 min. The nitrous acid treatment was performed for 2 h at room temperature. Control slides were placed in distilled water. After washing all sections three times in distilled water, they were placed overnight in alcian blue dissolved in solutions of 0.25, 0.55, 0.75 or 1.0 M magnesium chloride. Finally the sections were rinsed 5 min in flowing tap water and counterstained with neutral red. As control tissues, dog tracheal cartilage, skin, umbilical cord and connective tissue were included. Mast cells and intact basement membranes were used as internal controls.

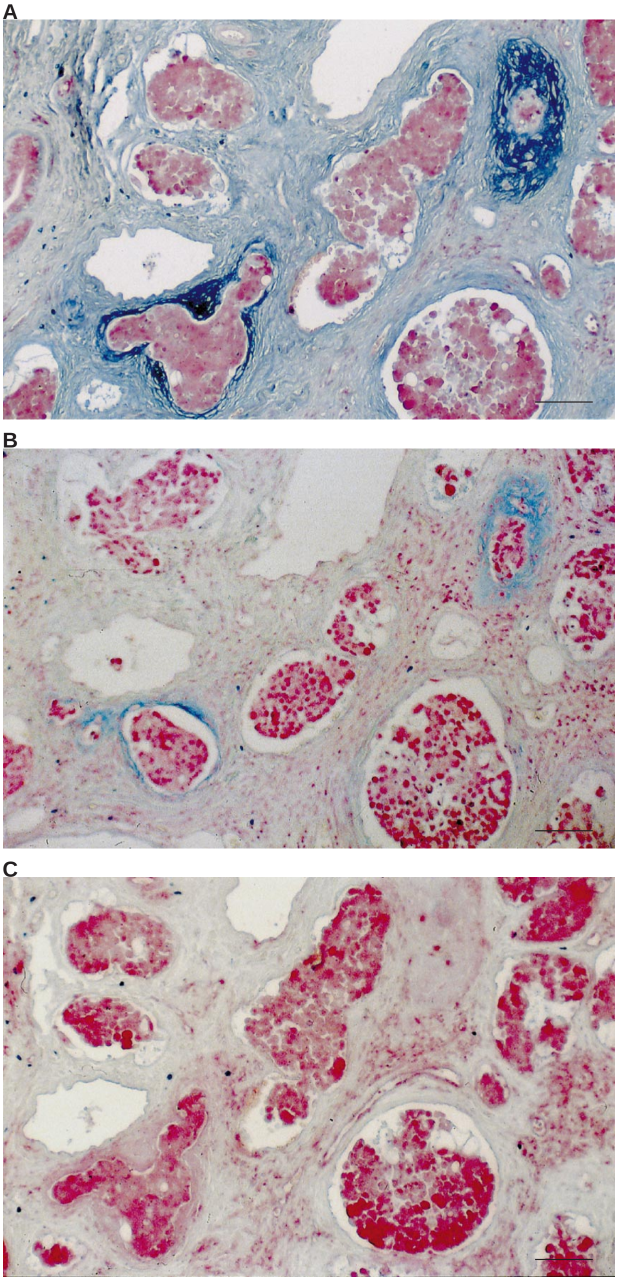

**Figure 1** Sulphated GAG accumulation as a mesenchymal reaction around clusters of tumour cells, in serial slides of an anaplastic carcinoma, stained with alcian blue pH 5.8. (**A**) Alcian blue with 0.75 M magnesium chloride. (**B**) Alcian blue with 1 M magnesium chloride. (**C**) Alcian blue with 1 M magnesium chloride after pretreatment with chondroitinase AC; mast cells with HS are indicated with arrows. Bar = 94 µm

The specificity of enzymes and nitrous acid was tested by degradation of s-GAGs (keratan sulphate, chondroitin sulphate A, chondroitin sulphate B/dermatan sulphate, heparan sulphate from Sigma, St Louis; heparin from Organon, Oss, The Netherlands) on cellulose acetate membranes.

Immunohistochemical confirmation of the presence and distribution of CS was obtained using murine monoclonal antibody (mAb) antichondroitin sulphate A and C (Biogenesis, Bournemouth, UK, clone-Nr CS-56, IgM) against the GAG part of PG and the murine monoclonal anti-PG ΔDi-6S IgM (3B3) (Seikagaku Corporation, Tokyo).

© 1999 Cancer Research Campaign

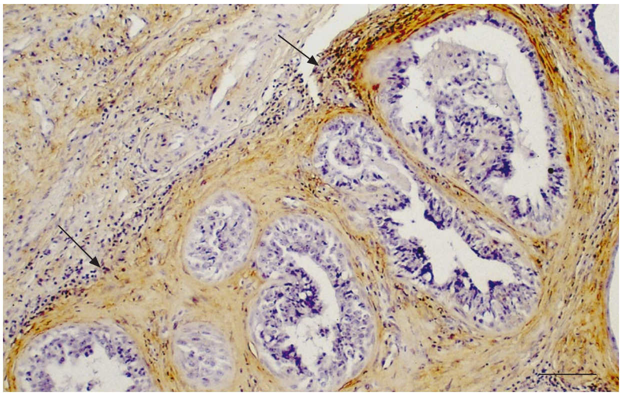

**Figure 2** Adenocarcinoma stained with monoclonal antibody against CS showing CS accumulation as a mesenchymal reaction around clusters of tumour cells (arrows). Immunoperoxidase. Bar = 94 µm

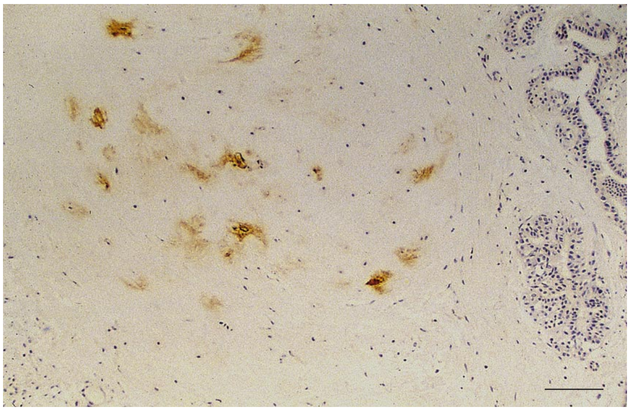

**Figure 4** Expression of C6-S in a benign mixed tumour shown by staining with the 3B3 monoclonal antibody. Immunoperoxidase. Bar = 94 µm

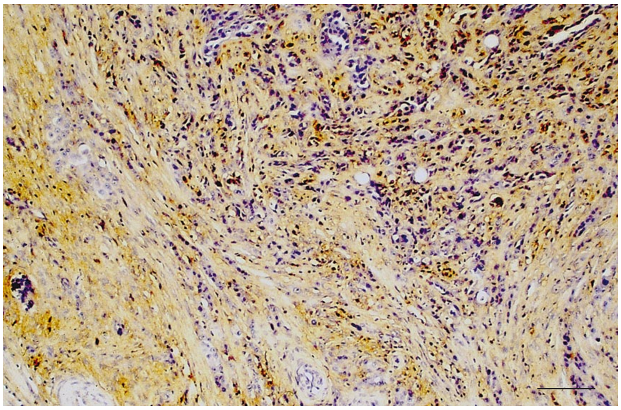

**Figure 3** Diffuse accumulation of CS between tumour cells in an adenocarcinoma stained with a monoclonal antibody against CS. Bar = 94 µm

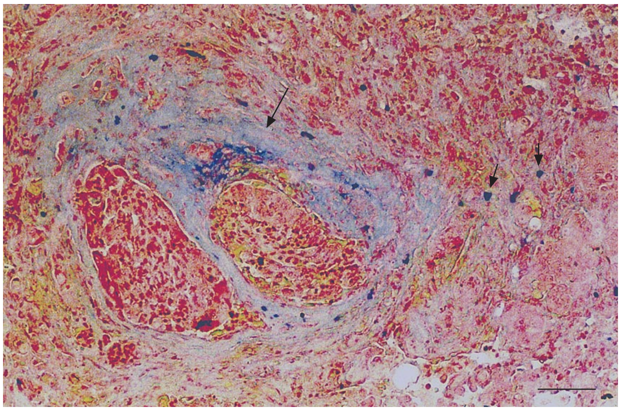

**Figure 5** Sulphated GAG accumulation (large arrow) as a mesenchymal reaction around clusters of tumour cells in a metatasis of a solid carcinoma in the lung. Some alcian blue positive mast cells are also visible (small arrows). Alcian blue pH 5.8, 1.0 M magnesium chloride. Bar = 62 µm

Immunohistochemistry with CS-56 mAb was performed, using an indirect peroxidase method with antibody dilutions of 1:400 for the primary antibody and 1:100 for the goat anti-mouse peroxidase labelled second antibody.

For immunoperoxidase staining with the 3B3 mAb, the indirect streptavidin–biotin method was used. The 3B3 mAb was applied on 12 cases representing all tumour types. This antibody reacts with the stub of ΔDi-6S units of chondroitin-6-sulphate (CS6-S) obtained from CSPG by chondroitinase ABC digestion. A native epitope is located at the non-reducing termini of CS6-S, present in embryonic and arthritic cartilage (Caterson et al, 1990). The primary antibody 3B3 was diluted 1:150, the second biotin-labelled rabbit anti-mouse antibody was used in a dilution of 1:500 and, finally, the slides were incubated with the avidin–biotin–peroxidase complex in a dilution of 1:100. In both cases, staining was visualized with diaminobenzidine and counterstained with Meyers haematoxylin.

A rabbit anti-chicken fibronectin polyclonal antibody (Chemicon International, Temecula, CA, USA) was used as a marker for the detection of the extracellular fibronectin. The polyclonal antibody was applied on slides pretreated for 30 min at room temperature with 0.05% saponin (Oncogene Sciences, Uniondale, NY, USA); slides were incubated overnight at 4°C diluted 1:2600. Staining was visualized with an indirect immunoperoxidase staining.

### RESULTS

Light-microscopic investigation of alcian blue-stained mammary gland tissue, including normal mamma, hyperplasias and tumours, revealed the presence of the four s-GAGs. As demonstrated by selective degradation procedures, HS and CS were the dominant GAGs; dermatan sulphate and keratan sulphate were only present in a limited number of cases in low amounts and are therefore not shown. Considerable accumulation of CS could be detected in the tumours by alcian blue. Its presence and distribution were confirmed by immunohistochemistry; although the immunohistochemistry is the more simple and rapid method, use of alcian blue with the critical electrolyte concentration permits the study of the degree of sulphation of the detected CS. In general, five patterns of s-GAG accumulation were found which will be described consecutively.

Most prominent was the accumulation of s-GAGs appearing as a mesenchymal reaction around nests and clusters of tumour cells (Figure 1A); it was identified as CS by disappearance of alcian blue staining after chondroitinase AC pretreatment of the sections

*© 1999 Cancer Research Campaign* 

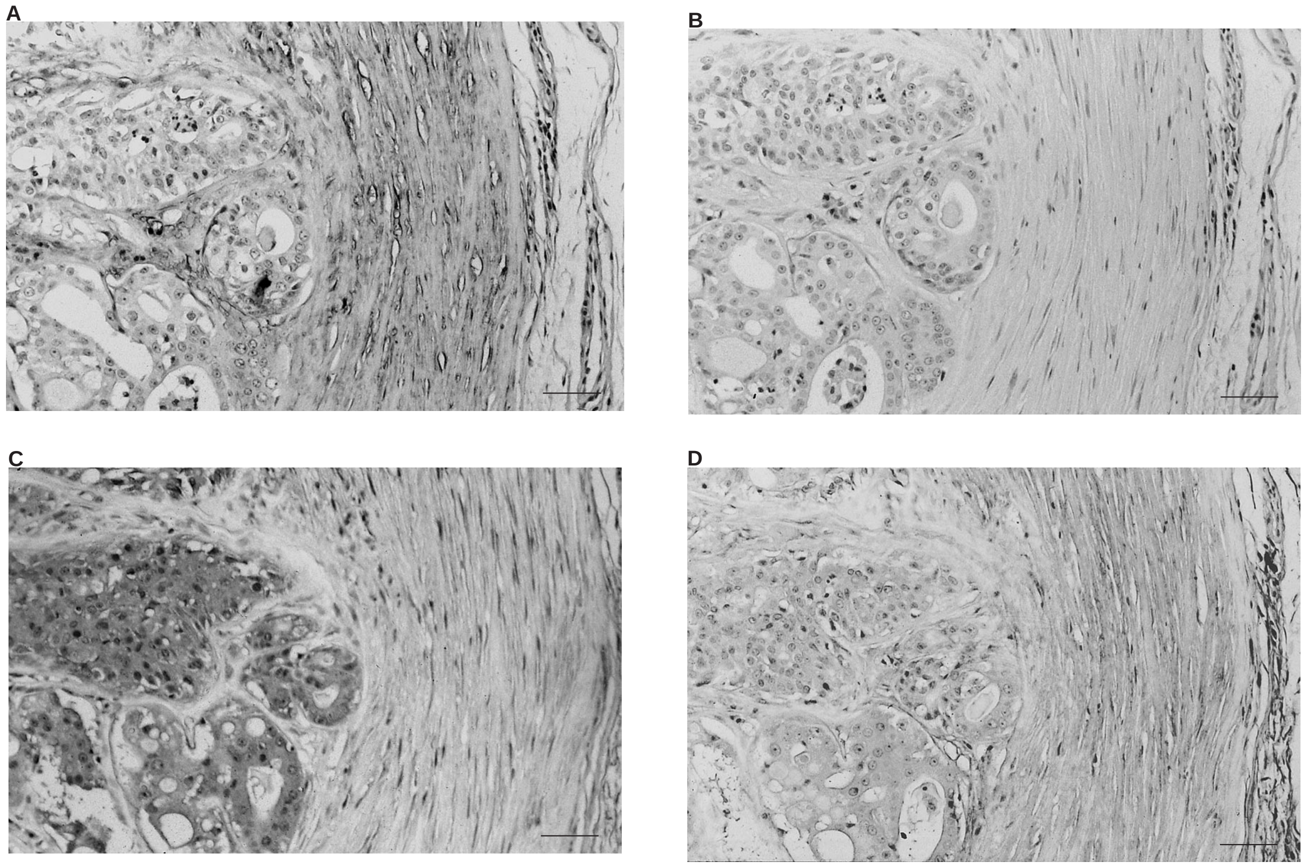

**Figure 6** Effect of chondroitin sulphate degradation on immunolocalization of fibronectin. Serial slides of a canine mammary complex adenoma with a stromal reaction were stained. (**A**) Chondroitin sulphate staining with a monoclonal antibody against CS. (**B**) As in **A**, but with pretreatment with chondroitinase AC. (**C**) Fibronectin staining with a rabbit polyclonal against fibronectin. (**D**) As in **C**, but with pretreatment with chondroitinase AC. Bar = 47 μm. Non-specific staining with the rabbit polyclonal is seen in the cytoplasm of the cells in **C** and **D**

(Figure 1C) and by immunohistochemistry with the CS-56 monoclonal antibody (Figure 2). In more than half of the specimens the s-GAG staining was maintained in alcian blue pH 5.8 and 1 M magnesium chloride, indicating a high sulphation of CS (Figure 1B). The staining was most intense in mesenchymal tissue closely interwoven with single tumour cells or clusters of cells, suggesting an epithelial-induced production of CS by fibroblasts. However, not all tumour–stroma junctions showed intense staining.

As a second pattern, diffuse s-GAG accumulation between tumour cells was observed and also characterized as CS by immunohistochemistry (Figure 3). In most cases, alcian blue and CS-56-positive structures were directly accumulating around luminal epithelial and myoepithelial cells, whereas fibroblasts could not be identified by morphological criteria (Figure 3). However, in some cases cells neighbouring s-GAGs were vimentin-positive (not shown). In complex tumours, diffuse accumulation of CS was very prominent in the myxoid areas with spindle cell proliferation.

In the third pattern of s-GAG accumulation, i.e. fibrillar alcian blue-positive structures, the s-GAGs had accumulated between acini or cords and clusters of normal and tumourous epithelial cells. They are likely to be basement membranes or remnants of former basement membrane structures, but this was not investigated further. The fibrillar structures contained a heterogeneous mixture of s-GAGs shown by nitrous acid and enzyme digestion; in addition to HS, CS could be demonstrated in it by CS-56 immunohistochemistry. A related type of s-GAG accumulation was visible as periductal thickening in seven of 86 mammary glands containing hyperplasias, adenomas and carcinomas. Characterization of s-GAGs in this structure by degradation with enzymes and acid revealed that HEP/HS and CS had accumulated here. A positive reaction with the mAb against CS was predominantly found at the basolateral side of this basement membrane-like structure.

The fourth pattern was classified as cartilaginous or myxoid s-GAG accumulation. It was characterized by matrix-rich areas with few polyhedral cells with a distinct halo. Alcian blue-positive staining in precartilaginous areas was absent after chondroitinase AC and ABC digestion. In dense cartilaginous areas with transition towards bone forming, alcian blue staining was only weakened, most likely because of keratan sulphate (KS) accumulation. In 1.0 M magnesium chloride the positive staining with alcian blue disappeared in precartilaginous areas, whereas in cartilage only perilacunar areas were positive. CS-56 antibody reacted positively in matrix, and pericellularly in cartilaginous as well as precartilaginous areas. However, staining in dense matrix of cartilage was very weak, whereas the perilacunar chondroid matrix reacted strongly.

To study in more detail the presence of cartilage-related CS, the 3B3 mAb was applied on 12 cases representing all tumour types; the CS6-S to which this antibody reacts is expressed in immature, but less so in mature cartilage (Caterson et al, 1990). The antibody

*© 1999 Cancer Research Campaign*

**Table 1** Stromal chondroitin sulphate (CS) accumulation in normal, hyperplastic and neoplastic mammary tissues of dogs and lung and lymph node metastases

| | | Mesenchymal CS accumulation | | Interepithelial diffuse CS matrix | |
|---|---|---|---|---|---|
| **Tissue type**[a] | **Total analysed** | **Total positive**[b] | **Highly sulphated**[c] | **Total positive** | **Highly sulphated** |
| Normal mammary gland | 4 | 0 | | 0 | |
| Hyperplasia | 7 | 0 | | 0 | |
| Simple adenoma | 2 | 0 | | 0 | |
| Complex adenoma | 20 | 12 | 10 | 15 | 7 |
| Benign mixed tumour | 10 | 6 | 3 | 9 | |
| Simple carcinoma | 20 | 18 | 14 | 1 | |
| Complex carcinoma | 8 | 7 | 4 | 6 | 2 |
| Malignant mixed carcinoma | 2 | 2 | 1 | 2 | |
| Anaplastic carcinoma | 12 | 10 | 5 | 1 | |
| Total tumours | 74 | 55 | 37 | 34 | 9 |
| Simple carcinoma lung metastases | 2 | 2 | 0 | | |
| Lymph node metastases | 2 | 2 | 1 | 2 | 1 |
| Anaplastic carcinoma lung metastases | 7 | 7 | 6 | 5 | 4 |
| Lymphe node metastases | 6 | 5 | 4 | 3 | 3 |
| Total metastases | 17 | 16 | 11 | 10 | 8 |

[a]Classification according to WHO criteria (Hampe and Misdorp, 1974). [b]Staining with alcian blue, pH 5.8; 0.25 M magnesium chloride. [c]Staining with alcian blue, pH 5.8; 1.00 M magnesium chloride.

stained the myxoid areas around single cells in mixed tumours. The positive reaction was most pronounced in matrix-rich areas containing just a few polyhedral cells (Figure 4); it was also seen around spindle-shaped cells in the myxoid matrix (not shown).

The fifth pattern of s-GAG accumulation was intracellular storage of s-GAGs in mast cells, which was visible in almost all primary tumours (Figure 1) and metastases (Figure 5). The number of mast cells was increased in tumours and hyperplasias compared to the normal tissue, with exception of some simple and anaplastic carcinomas. Intracellularly, stored s-GAGs could be identified as HEP/HS by pretreatment with nitrous acid. However, in two cases (anaplastic carcinoma and complex carcinoma) a few mast cells were still alcian blue-positive after pretreatment with nitrous acid. These may represent mast cells of the mucosal type, that are known to contain CS (Abbas et al, 1991).

Investigation of metastatic lesions of mammary tumours in lung and lymph node revealed essentially the same patterns of s-GAG accumulation as those in primary tumours, with the mesenchymal s-GAG accumulation around clusters of tumour cells as the most prominent feature (Figure 5). This pattern was most striking around metastatic emboli in vessels. The only s-GAG identified in the metastases is CS. As in the primary tumours some metastases showed alcian blue-positive structures at 1.0 M magnesium chloride, characterized as highly sulphated CS.

The distribution of the different patterns of s-GAG accumulation in the mammary tissues analysed for this study is given in Table 1 with regard to the first and the second pattern, i.e. mesenchymal CS accumulation and diffuse interepithelial CS accumulation. CS accumulation in a mesenchymal pattern around clusters of tumour cells (Figure 1 and 2) was detectable in all types of benign and malignant primary tumours, except in the only two simple adenomas that were analysed. The actual numbers are 55 positive tumours out of 74 studied (74%). Highly sulphated CS was present in 37 primary tumours, i.e. 50% of the total number and 67% of CS-containing tumours with this pattern (Table 1). As described above, a similar pattern of CS accumulation was found in metastases in lung and lymph node, derived from three anaplastic and 11 simple carcinomas (Table 1). The total number of our investigated primary tumours giving rise to metastases with highly sulphated CS was not determined.

The second pattern, i.e. the diffuse CS accumulation, was also absent in normal and hyperplastic tissues; its presence in the tumours seems to be related to tumour types. It was found in 34 tumours (46% of total) of which 32 were of complex and mixed types; in contrast, in simple adenomas, simple carcinomas and anaplastic carcinomas this type of CS accumulation was rare (two out of 34), indicating that its presence may be related to the activity of spindle and cartilage cells in the complex and mixed tumours.

The distribution of the remaining patterns is not shown in Table 1, because of their widespread occurrence. The pattern of fibrillar s-GAG accumulation was present in all normal and benign tissues, but in malignant tissues it was present in 26 out of 42 cases only, underlining their basement membrane-like nature. The pattern of cartilaginous/myxoid s-GAG accumulation was only found in so-called mixed tumours. The fifth pattern, accumulation of s-GAG-containing mast cells, was a general phenomenon and occurred in nine out of 11 normal glands and hyperplasias, in 32 out of 32 benign tumours and in 35 out of 42 malignant tumours.

Of the tumours, 19 specimens were studied for the localization of fibronectin in the stroma. After predigestion of the slides with saponin only, the fibronectin was found almost ubiquitous and equally distributed throughout the ECM of the tumours, except in areas with increased amounts of CS (made visible with either alcian blue staining or by immunohistochemistry). By preincubation of the slides with chondroitinase AC or ABC fibronectin staining was considerably increased. An example of fibronectin and CS staining of tumour stroma and the effects of chondroitinase AC pretreatment is shown in Figure 6.

### DISCUSSION

Based on the findings presented here, it can be concluded that CS at different localizations and with different sulphation is the predominant s-GAG in mammary tumours of dogs. CS has been

© 1999 Cancer Research Campaign described as an important s-GAG of the matrix in many epithelial tumours (Iozzo, 1985*a*), and elevated amounts of CS in human breast carcinomas have been demonstrated (Alini and Losa, 1991). However, their highly sulphated nature has not been shown before. The type of proteoglycan responsible for this CS accumulation is not known. The only tumour-specific PG characterized so far is the versican-related PG of Nara et al (1997). An early study on GAG distribution in mammary tumours of dogs, focusing on the origin of cartilage and precartilaginous matrix and complex tumours, claimed that hyaluronic acid is the primary GAG in these tumours (Palmer and Monlux, 1979). In the present study, CS could be identified as being the predominent s-GAG in these areas. However, hyaluronic acid, an unsulphated GAG, may be present as well, but was not included in our investigations.

HS was found only in pre-existing basement membranes, in remnants of it, or in apparently newly formed basement membrane fragments in metastases; in addition, it was present in mast cells, probably mixed with heparin.

KS has been found as constituent of the ECM in pleomorphic salivary gland tumours of humans (Nara et al, 1991) that show some similarities to complex and mixed mammary tumours of dogs with proliferation of the luminal and myoepithelial cells. In the present study, KS was presumably a constituent of alcian blue-positive structures in fibrillar depositions between epithelial cells as well as in cartilaginous areas of mixed tumours.

Detectable amounts of dermatan sulphate could not be identified with certainty in the tumour tissues. Fibrillar accumulation of s-GAGs between epithelial cells in primary tumours revealed a heterogeneous mixture of s-GAGs, in which dermatan sulphate might also be present.

### The pattern of mesenchymal CS accumulation around clusters of tumour cells

Although the incidence of CS accumulation in the stroma surrounding clusters and nests of tumour cells was found to be higher in the group of the malignant tumours than in benign tumours its presence cannot be considered as a marker for malignancy. This pattern was evident in metastatic lesions as well and was in these cases frequently seen around metastatic emboli. The importance of s-GAGs for establishing normal duct structures in developing mammary tissue by channelling end-bud growth has been pointed out (Silberstein and Daniel, 1982). At the electron microscopical level s-GAG accumulation has been identified in lactating human mammary tissue at several sites (Erlinger et al, 1990). This led to the conclusion, that these s-GAGs may be involved in cell differentiation or changes of breast tissue during lactation. On the other hand, new formation of basement membranes or desmoplastic connective tissue may form an additional barrier for infiltrating tumour cells.

Depositions of CS at the tumour–stroma junction were found by us to be often highly sulphated. Earlier, highly, i.e. trisulphated, CS disaccharides were previously described in dog mastocytoma cell lines (Forsberg et al, 1988), but were, up to now, only found intracellularly.

Furthermore, it was shown by in vitro studies, that the degree of sulphation of polysaccharides added to the media of cultured cells is correlated with the degree of inhibition of SV-T2 tumour cell growth (Klebe et al, 1986). Thus, highly sulphated CS together with desmoplastic changes as they occur in primary tumour lesions and in metastatic lesions may be the expression of an effort of the host tissue to inhibit tumour growth. Other ultrastructural alterations of PG in connective tissue of infiltrating carcinomas compared to normal mammary gland have also been reported (Losa and Alini, 1993). Fibroblasts at the tumour–stroma junctions may be responsible for the high amounts of CS. In vitro studies of other groups already have shown tumour cell-induced production of CS by fibroblasts (Takeuchi et al, 1976; Iozzo, 1985*b*; Merrilees and Finlay, 1985; Edward et al, 1992).

### The pattern of diffuse interepithelial CS accumulation

This pattern of CS accumulation was mainly confined to complex and mixed benign and malignant tumours. The main characteristic of the complex tumours is the presence of spindle cells; these are thought to be derived from myoepithelial cells and may give rise to metaplastic cartilage in canine mammary tumours (Pulley, 1973), although the myoepithelial origin of the spindle cells has been debated in favour of a stromal origin (Vos et al, 1993*a*, 1993*b*). Our findings with regard to GAG accumulation support the spindle cell origin of the cartilage in mixed tumours. First, the absence of the diffuse CS accumulation in tumours other than the complex and mixed types suggests the latter types to form a distinct group of tumours. Second, the 3B3 epitope, which is transiently expressed during definite stages of cellular differentiation and development of cartilage (Caterson et al, 1990), is detected in the matrix surrounding spindle cells as well as cartilage, suggesting that cartilage formation in mixed tumours is sequential to the formation of myxoid, precartilaginous tissue by the spindle cells in complex tumours.

### Other patterns

Fibrillar accumulation of s-GAGs between epithelial cells, the third pattern of s-GAG accumulation described above, occurred in almost all glandular structures, but staining intensity with CS-56 as well as with alcian blue was stronger in neoplastic lesions. Although these stromal components may represent basement membrane-related structures because of morphological criteria and the identification of HS in it, its identification awaits further confirmation by immunohistochemistry of laminin and collagen IV. The synthesis of new fragments of basement membranes by breast carcinomas, both in primary tumours and metastases, has been described (Albrechtsen et al, 1986; Guelstein et al, 1993).

The cartilage found in mixed benign and malignant tumours contains normal chondrocytes, as has been assessed by ultrastructural criteria (Pulley, 1973). Since highly sulphated CS was not found in the mature cartilage of these tumours, as it was in the areas of spindle cell proliferation, we propose that during the differentiation of spindle cells to cartilage the expression of the highly sulphated CS is lost.

The role of mast cells, present in increased numbers in tumours and hyperplasias of dog mammary tissue, remains unclear. In the literature their pathogenetic role in tumours is discussed controversially as ranging from a defensive role as participant in inflammatory processes to tumour promoting effects by stimulation of angiogenesis and tumour cell proliferation (Roche, 1986).

In conclusion, in dogs CS is an important component of the stroma of mammary tumours. This may reflect a more general phenomenon as it has been found in human breast cancer (Alini and Losa, 1991) and colon cancer and its metastases (Yamori et al, 1988). The nature of the proteoglycan involved is unknown, but next to the earlier mentioned versican, decorin is a likely candidate

© 1999 Cancer Research Campaign

(Adany et al, 1990). Whether these proteoglycans also provide the core protein for highly sulphated CS chains remains to be studied.

Clues to the biological function of CS accumulation in tumours come from our finding of CS-related decreased accessibility of fibronectin epitopes for antibodies (Figure 6). From in vitro studies it had already been concluded that the immobilized large CSPG from chicken fibroblasts, the versican-like protein of Nara et al (1997), was shown to inhibit the adhesion of cells to fibronectin (Yamagata et al, 1989). Our results now suggest that such an interaction between CSPGs and fibronectin may also exist in tumours in situ and thus may contribute to malignant behaviour of tumour cells in tissues. A similar effect of CS degradation was observed for immunohistochemistry of tenascin (not shown).

### ACKNOWLEDGEMENTS

We wish to thank Dr MHF van Niel for her assistance in histological diagnosis of the tumours, Dr RH Ostendorf (Jan van Breemen Institute, Amsterdam) for performing the 3B3 immunostaining, JA van Drie, RF Molenbeek and PCJ Tooten for their technical assistance and Dr W Misdorp for valuable advice. This work was supported by a grant from the ‘Evangelische Studienwerk’ to UH and by the ‘Legaat-Zwart’ for the Stimulation of Oncological Research.

### REFERENCES

Abbas KA, Lichtman AH and Pober JS (1991) *Cellular and Molecular Immunology*, p. 289. WB Saunders: Philadelphia

Adany R, Heimer R, Caterson B, Sorrell JM and Iozzo RV (1990) Altered expression of chondroitin sulphate proteoglycan in the stroma of human colon carcinoma. Hypomethylation of PG-40 gene correlates with increased PG-40 content and mRNA. *J Biol Chem* **265**: 11389–11396

Albrechtsen R, Wewer UM and Liotta LA (1986) Basement membranes in human cancer. *Pathol Annu* **51**: 251–276

Alini M and Losa GA (1991) Partial characterization of proteoglycans isolated from neoplastic and nonneoplastic human breast tissue. *Cancer Res* **51**: 1443–1447

Andres JL, Stanley K, Cheifetz S and Massagué J (1989) Membrane-anchored and soluble forms of betaglycan, a polymorphic proteoglycan that binds transforming growth factor-β. *J Cell Biol* **109**: 3137–3145

Caterson B, Mahmoodian F, Sorrell JM, Hardingham TE, Bayliss MT, Carney SL, Ratcliffe A and Muir H (1990) Modulation of native chondroitin sulphate structure in tissue development and in disease. *J Cell Sci* **97**: 411–417

David G (1993) Integral membrane heparan sulfate proteoglycans. *FASEB J* **7**: 1023–1030

Edward M, Grant AW and Mackie RM (1992) Human melanoma cell-derived factor(s) stimulate fibroblast glycosaminoglycan synthesis. *Int J Cancer* **52**: 499–503

Erlinger R, Schumacher U and Welsch U (1990) Ultrastructural localisation of glycosaminoglycans in the human mammary gland. *Acta Histochem* **Suppl. XL**: 65–70

Forsberg LS, Lazarus SC, Seno N, Devinney R, Caughey GH and Gold WM (1988) Dog mastocytoma proteoglycans: occurrence of heparin and oversulphated chondroitin sulphates, containing trisulphated disaccharides, in three cell lines. *Biochim Biophys Acta* **967**: 416–428

Guelstein VI, Tchypysheva TA, Ermilova VD and Ljubimov AV (1993) Myoepithelial and basement membrane antigens in benign and malignant human breast tumours. *Int J Cancer* **53**: 269–277

Hampe JF and Misdorp W (1974) Tumors and dysplasias of mammary gland. *Bull Wld Hlth Org* **50**: 111–133

Iozzo RV (1985*a*) Proteoglycans: structure, function and role in neoplasia. *Lab Invest* **53**: 373–392

Iozzo RV (1985*b*) Neoplastic modulation of extracellular matrix: colon carcinoma cells release polypeptides that alter proteoglycan metabolism in colon fibroblasts. *J Biol Chem* **260**: 7464–7473

Iozzo RV (1987) Proteoglycans and the intercellular tumor matrix. In *Current Topics in Pathology*, Seifert G (ed), pp. 207–221. Springer: Berlin

Iozzo RV (1988) Proteoglycans and neoplasia. *Cancer Metast Rev* **7**: 39–50

Iozzo RV (1997) The family of the small leucine-rich proteoglycans: key regulators of matrix assembly and cellular growth. *Crit Rev Biochem Mol Biol* **32**: 141–174

Jackson RL, Busch SJ and Cardin AD (1991) Glycosaminoglycans: molecular properties, protein interaction and role in physiological processes. *Physiol Rev* **71**: 481–539

Klebe RJ, Escobedo LV, Bentley KL and Thompson LK (1986) Regulation of cell motility, morphology, and growth by sulphated glycosaminoglycans. *Cell Motil* **6**: 273–281

Losa GA and Alini M (1993) Sulphated proteoglycans in the extracellular matrix of human breast tissues with infiltrating carcinoma. *Int J Cancer* **54**: 552–557

Merrilees MJ and Finlay GJ (1985) Human tumor cells in culture stimulate glycosaminoglycan synthesis by human skin fibroblasts. *Lab Invest* **53**: 30–36

Misdorp W and Hart AAM (1976) Prognostic factors in canine mammary cancer. *J Natl Cancer Inst* **56**: 779–786

Nara Y, Kato Y, Torii Y, Tsuji Y, Nakagaki S, Goto S, Isobe H, Nakashima N and Takeuchi J (1997) Immunohistochemical localization of extracellular matrix components in human breast tumours with special reference to PG-M/versican. *Histochem J* **29**: 21–30

Nara Y, Takeuchi J, Yoshida K, Fukatsu T, Nagasaka T, Kawaguchi T, Meng N, Kikuchi H and Nakashima N (1991) Immunohistochemical characterisation of extracellular matrix components of salivary gland tumors. *Br J Cancer* **64**: 307–314

Niewold TA, Flores Landeira JM, Van den Heuvel LPWJ, Ultee A, Tooten PCJ and Veerkamp JH (1991) Characterization of proteoglycans and glycosaminoglycans in bovine renal AA-type amyloidosis. *Virchows Arch B Cell Pathol Mol Pathol* **60**: 321–328

Palmer TE and Monlux AW (1979) Acid mucopolysaccharides in mammary tumors of dogs. *Vet Pathol* **16**: 493–509

Pulley LT (1973) Ultrastructural and histochemical demonstration of myoepithelium in mixed tumors of the canine mammary gland. *Am J Vet Res* **34**: 1513–1522

Roche WR (1986) The nature and significance of tumor-associated mastcells. *J Pathol* **148**: 175–182

Roskelley CD and Bissell MJ (1995). Dynamic reciprocity revisited: a continuous, bidirectional flow of information between cells and the extracellular matrix regulates mammary epithelial cell function. *Biochem Cell Biol* **73**: 391–397

Santra M, Mann DM, Mercer EW, Skorski T, Calabretta B and Iozzo RV (1997) Ectopic expression of decorin protein core causes generalized growth suppression in neoplastic cells of various histogenetic origin and requires endogenous p21, an inhibitor of cyclin-dependent kinases. *J Clin Invest* **100**: 149–157

Scott JE and Dorling J (1965) Differential staining of acid glycosaminoglycans (mucopolysaccharides) by alcian blue in salt solutions. *Histochemistry* **5**: 221–233

Silberstein-GB and Daniel CW (1982) Glycosaminoglycans in the basal lamina and extracellular matrix of developing mouse mammary duct. *Dev Biol* **90**: 215–222

Takeuchi J, Sobue M, Sato E, Shamoto M, Suzuki S and Kimata K (1976) Variation in glycosaminoglycan components of breast tumours. *Cancer Res* **36**: 2133–2139

Vos JH, Van den Ingh TSGAM, Misdorp W, Molenbeek RF, Van Mil FN, Rutteman GR, Ivanyi D and Ramaekers FCS (1993*a*) Immunohistochemistry with keratin, vimentin, desmin and α-smooth muscle actin monoclonal antibodies in canine mammary gland: benign mammary tumours and ductectasias. *Vet Quart* **15**: 89–95

Vos JH, Van den Ingh TSGAM, Misdorp W, Molenbeek RF, Van Mil FN, Rutteman GR, Ivanyi D and Ramaekers FCS (1993*b*) Immunohistochemistry with keratin, vimentin, desmin and α-smooth muscle actin monoclonal antibodies in canine mammary gland: malignant mammary tumours. *Vet Quart* **15**: 96–101

Yamagata M, Suzuki S, Akiyama SK, Yamada M and Kimata K (1989) Regulation of cell-substrate adhesion by proteoglycans immobilized on extracellular substrates. *J Biol Chem* **264**: 8012–8018

Yamori, T, Ota DM, Cleary KR and Irimura T (1988) Increased content of chondroitin sulphate proteoglycan in human colorectal carcinoma metastases compared with the primary tumor as determined by an anti-chondroitin-sulphate monoclonal antibody. *J Cell Biochem* **36**: 405–416

© 1999 Cancer Research Campaign
