# Erratum

**DOI:** 10.1038/sj.bjc.6690613

**Published:** 1999-08

**Authors:** U Hinrichs, G R Rutteman, H Nederbragt


					Stromal accumulation of chondroitin sulphate in
mammary tumours of dogs
U Hinrichs, GR Rutteman and H Nederbragt
Br J Cancer 80(9): 1359-1365
Figure 6 is reproduced correctly below:
Erratum
1866
British Journal of Cancer (1999) 80(11), 1866
(c) 1999 Cancer Research Campaign
Article no. bjoc.1999.0613
A B
C D

				

## 

**Correction to:** *British Journal of Cancer* (1999) **80**, 1359–1365. doi:10.1038/sj.bjc.6690529

